# Bayesian Learning-Based Clustered-Sparse Channel Estimation for Time-Varying Underwater Acoustic OFDM Communication †

**DOI:** 10.3390/s21144889

**Published:** 2021-07-18

**Authors:** Shuaijun Wang, Mingliu Liu, Deshi Li

**Affiliations:** 1 Electronic Information School, Wuhan University, Wuhan 430072, China; wangsj@whu.edu.cn (S.W.); liumingliu@whu.edu.cn (M.L.); 2 Collaborative Innovation Center of Geospatial Technology, 129 Luoyu Road, Wuhan 430072, China

**Keywords:** clustered-sparse channel estimation, Bayesian learning, underwater acoustic communication, orthogonal frequency division multiplexing

## Abstract

Orthogonal frequency division multiplexing (OFDM) has been widely adopted in underwater acoustic (UWA) communication due to its good anti-multipath performance and high spectral efficiency. For UWA-OFDM systems, channel state information (CSI) is essential for channel equalization and adaptive transmission, which can significantly affect the reliability and throughput. However, the time-varying UWA channel is difficult to estimate because of excessive delay spread and complex noise distribution. To this end, a novel Bayesian learning-based channel estimation architecture is proposed for UWA-OFDM systems. A clustered-sparse channel distribution model and a noise-resistant channel measurement model are constructed, and the model hyperparameters are iteratively optimized to obtain accurate Bayesian channel estimation. Accordingly, to obtain the clustered-sparse distribution, a partition-based clustered-sparse Bayesian learning (PB-CSBL) algorithm was designed. In order to lessen the effect of strong colored noise, a noise-corrected clustered-sparse channel estimation (NC-CSCE) algorithm was proposed to improve the estimation accuracy. Numerical simulations and lake trials are conducted to verify the effectiveness of the algorithms. Results show that the proposed algorithms achieve higher channel estimation accuracy and lower bit error rate (BER).

## 1. Introduction

Underwater acoustic (UWA) sensor networks can be applied to a variety of underwater activities, including environment monitoring, resource exploration and equipment navigation. Reliable high-rate communication is significant for massive data transmission between sensor nodes [1]. However, the UWA channel is widely known as one of the most difficult communication media, and high-rate communication with high reliability is quite challenging. Since the sound attenuation rapidly increases with frequency, the bandwidth available for communication is extremely limited. Due to the reflection and refraction of sound in the ocean, the multipath spread in a medium-range shallow water channel can extend over tens or even hundreds of milliseconds [2], resulting in severe frequency-selective signal distortion. Due to the dynamic environment and moving transceivers in ocean, the UWA channel is time-varying. In particular, the normalized frequency offset factor induced by Doppler can be on the order of $10^{-3}$ compared with $10^{-7}$ for high-speed mobile radio channels [2].

**Figure 1:**
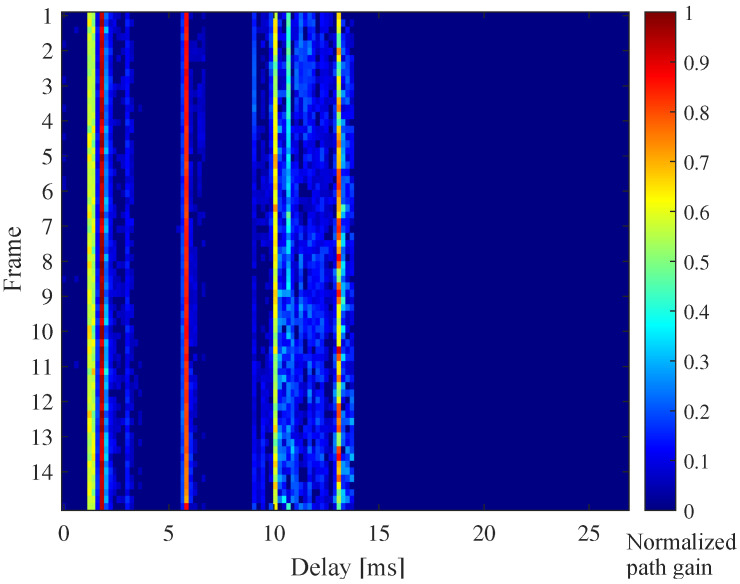
Time-varying multipath channel with clustered-sparse structure estimated in a lake communication trial.

Due to high spectral efficiency and good anti-multipath performance, orthogonal frequency division multiplexing (OFDM) and some OFDM-based modulation techniques are widely adopted for UWA communications [3,4,5,6]. In UWA-OFDM systems, accurate channel state information (CSI) is vital for channel equalization and adaptive transmission [7,8], which can significantly affect the reliability and throughput. However, it is difficult to estimate the time-varying channel impulse responses (CIRs) with excessive delay spread. To overcome this problem, some structural information such as sparsity can be exploited to improve channel estimation performance. The paths of the UWA channel tend to be clustered-sparse, which carry comprehensive structural information [9,10,11,12,13]. As shown in Figure 1, there are many weak paths centering around the eigen-paths associated with the medium refractions and surface/bottom bounces, which form the clustered-sparse structure. As different clusters are usually derived from different transmission routes, the channel coherence time usually varies across clusters. Thus, the path variation for different clusters can reflect the channel variation characteristics [14], and can be used for predicting the time-varying UWA channel [15]. However, the fading process of the UWA channel may randomly vary [2], which makes it difficult to accurately obtain the path variation for different clusters between adjacent OFDM blocks. To obtain the clustered-sparse structure of the UWA channel, we proposed a clustered-sparse Bayesian channel estimation method in [16], which is the preliminary version of this work (This work extends the conference paper [16]) by (i) adding a Bayesian learning-based channel estimation architecture; (ii) adding a noise-corrected clustered-sparse channel estimation algorithm to lessen the effect of strong colored noise; (iii) giving the detailed derivation and performance analysis of the proposed algorithms; and (iv) presenting more complete performance results related to modulation order, model hyperparameters and noise distribution).

In addition to the time-varying CIRs with excessive delay spread, the complex distribution of noise in the UWA channel also makes the channel estimation difficult. Generally, there are two kinds of channel noise, including the ambient noise and site-specific noise. The former type is presented in the background of the quiet deep sea, and exhibits a colored characteristic when approximated by Gaussian distribution, while for the site-specific noise, significant non-Gaussian components should be carefully considered [2]. Some experiments have confirmed that the noise is non-uniform across different locations, times and frequencies [17]. As a popular noise model in most UWA-OFDM systems, additive white Gaussian noise (AWGN) is straightforward for modeling and solving the noise distribution of the UWA channel, but it deviates from the actual underwater noise. Obviously, the deviation of the noise model means the estimation error of the noise distribution, which will lead to the degradation of channel estimation and signal detection performance.

To tackle the challenge of estimating the time-varying UWA channel with excessive delay spread and complex noise distribution, we adopted Bayesian learning to obtain the multipath and noise distribution characteristics of the UWA channel, based on which Bayesian channel estimation is derived to improve the performance of pilot-based channel estimation. The main contributions of this work are summarized as follows:

- To estimate the time-varying multipath channel with colored noise, we propose a novel Bayesian learning-based channel estimation architecture for UWA-OFDM systems. Specifically, a clustered-sparse channel distribution model is constructed to characterize the delay power spectrum and temporal correlation of each cluster in the multipath channel, and a noise-resistant channel measurement model is constructed to reduce the noise disturbance. By learning the model hyperparameters, the Bayesian channel estimation based on the two models can be iteratively optimized.
- To obtain the clustered-sparse distribution, we propose a partition-based clustered-sparse Bayesian learning (PB-CSBL) algorithm. Through the cluster partition, different clusters can learn different channel correlation coefficients, and thus the inter-cluster interference of the multipath channel can be suppressed.
- To lessen the effect of strong colored noise, we propose a noise-corrected clustered-sparse channel estimation (NC-CSCE) algorithm. Based on the iterative symbol decision and noise correction, the more accurate hyperparameters of the models can be obtained, which can improve the accuracy of the Bayesian channel estimation.

The rest of this paper is organized as follows. Section 2 discusses the related works. In Section 3, the system architecture and channel models are constructed. Then, the Bayesian learning-based clustered-sparse channel estimation method is designed in Section 4. In Section 5, the noise-corrected clustered-sparse channel estimation method is presented. The evaluation and result analysis are shown in Section 6. In Section 7, conclusions are given.

## 2. Related Works

The UWA channel is severely band-limited, and exhibits time-varying excessive delay spread. To this end, the sparse structure of the UWA channel can be exploited to improve the estimation performance, as most channel energy of the UWA channel is concentrated on a few paths [18,19]. The paths of the UWA channel also tend to be clustered-sparse [20], which has been frequently observed based on field experiments at different locations [9,10,11,12,13]. A detailed investigation on the variation of the significant paths in each cluster is presented in [21]. Based on the observation of channel sparsity, compressed sensing (CS)-based sparse channel estimation methods [22,23,24] have been studied, such as the greedy matching pursuit (MP) and orthogonal matching pursuit (OMP), which were adopted in [18,25], aiming to estimate the UWA channel impulse response (CIR) and the channel delay-Doppler-spread function, respectively. In order to reduce the convergence error of the OMP algorithm, the compressed sampling matching pursuit (CoSaMP) algorithm is proposed in [26]. However, CoSaMP-based channel estimators require knowledge of the channel sparsity level, which is difficult to obtain accurately in advance in practical applications. The joint sparsity among adjacent OFDM blocks was exploited by the simultaneous OMP (SOMP) [27] for higher estimation accuracy, but only stable path delays were considered and the temporal correlation of path gains was ignored. Typical convex optimization algorithms including basis pursuit (BP) and basis pursuit denoising (BPDN) tackle the NP-hard $l_{0}$ norm problem by transforming the $l_{0}$ norm to a convex $l_{1}$ norm. The BP algorithm was applied to estimate the path delays and Doppler scale factors of the UWA channel in [25], and the BPDN algorithm was proposed to estimate the slowly time-varying UWA channels in [28]. However, the global minimum of the cost function in BP or BPDN is not necessarily consistent with the sparsest solution. An empirical channel variation model, in which different clusters vary independently, was proposed in [14], and demonstrated that adaptation with multiple clusters outperforms that with one cluster for UWA channel estimation.

In recent years, Bayesian learning (BL) has been used to improve the performance of channel estimation. Compared with the CS-based methods that provide point estimation, Bayesian techniques can give the interval estimation of channel response and have a desirable property of preventing structural errors [29]. By using BL-based methods, the high accuracy of channel estimation was achieved in OFDM systems [30,31,32,33]. The temporal multiple sparse Bayesian learning (TMSBL)-based estimation method was further proposed in [32] to estimate the sparse channels by taking advantage of the channel coherence between consecutive OFDM blocks, and it can achieve better performance in strong correlated channels and maintains robustness in weak temporal correlated channels. Due to the high computational complexity of BL, some accelerated algorithms were proposed, such as the approximate message passing (AMP)-based method in [34]. More recently, some researchers utilized deep learning (DL)-based channel estimators for OFDM systems [35,36,37], which showed better channel estimation or equalization performance for nonlinear channel distortion. However, how to go about improving the generalization performance of pre-training DL models and reduce the complexity of training and inferring remain challenging.

## 3. System Architecture and Channel Models

In this section, the system architecture of Bayesian learning-based channel estimation is presented first. Then, the noise-resistant channel measurement model and the clustered-sparse channel distribution model are constructed.

### 3.1. Bayesian Learning-Based Channel Estimation Architecture

**Figure 2:**
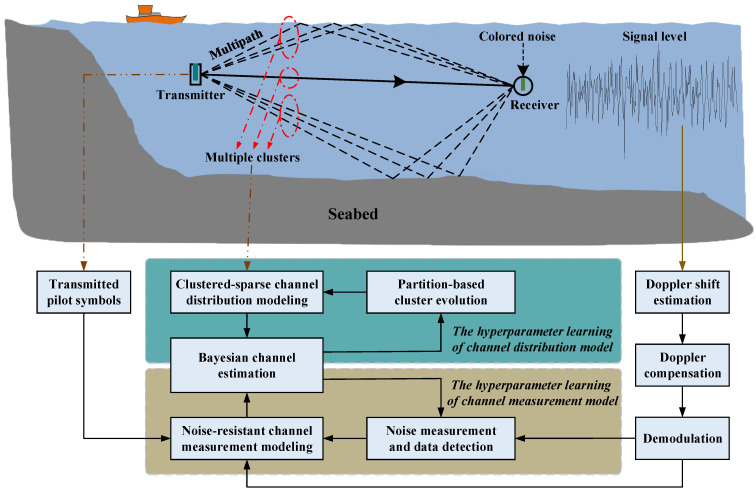
Architecture of Bayesian learning-based channel estimation in UWA-OFDM communication.

A typical UWA-OFDM communication scenario was shown at the top of Figure 2. The receiver can obtain the transmitted OFDM signal through dynamic multipaths, and the received signal may be affected by random colored noise. At the bottom of Figure 2, the system architecture of Bayesian learning-based channel estimation is given. To characterize the delay power spectrum and temporal correlation of each cluster in the UWA channel, the clustered-sparse channel distribution model is constructed and the model hyperparameters can be optimized by the iterative partition-based cluster evolution. Demodulated signal and pilot symbols were utilized to measure the instantaneous channel response, and the iterative noise measurement and data detection can improve the anti-noise performance of the channel measurement model. Based on the channel distribution model and the channel measurement model, the Bayesian channel estimation can be obtained.

Important notations used in following sections are given by:

- Uppercase and lowercase bold symbols are reserved for matrices and vectors, respectively. Particularly, $I_{M}$ denotes the identity matrix with size M×M. When the dimension is evident from the context, for simplicity, we only used I;
- $A^{T}$ and $A^{H}$ represent the transpose and conjugate transpose of A, respectively;
- A⊗B denotes the Kronecker product of the two matrices A and B;
- vec(A) denotes the vectorization of A formed by stacking its columns into a single column vector;
- Tr(A) denotes the trace of A;
- $Toeplitz(a_{1},a_{2},\ldots ,a_{N})$ denotes a Toeplitz matrix taking $a_{1},\ldots ,a_{N}$ as first row;
- diag(a) or $diag(a_{1},a_{2},\ldots ,a_{N})$ denotes a diagonal matrix with principal diagonal elements being $a_{1},a_{2},\ldots ,a_{N}$ in turn; $diag(A_{1},A_{2},\ldots ,A_{N})$ denotes a block diagonal matrix with principal diagonal blocks being the square matrices $A_{1},\ldots ,A_{N}$ in turn;
- If A is a square matrix, Prdiag(A) denotes a diagonal matrix with principal diagonal elements being the principal diagonal elements of A in turn.
- If some terms in a cost function do not contribute to the subsequent optimization of the parameters, ∝ is used to indicate that these terms have been dropped.

### 3.2. Noise-Resistant Channel Measurement Model

Considering the OFDM system with cyclic prefix (CP) and *K* subcarriers, the non-overlapping sets of $K_{d}$ data subcarriers $x_{d}$, $K_{p}$ pilot subcarriers $x_{p}$ and $K_{n}$ null subcarriers $x_{n}$ satisfy $K=K_{d}+K_{p}+K_{n}$. Let *T* denotes the OFDM symbol period without CP, $T_{cp}$ denotes the length of CP and $T_{s}=T+T_{cp}$ is the whole OFDM symbol period. The *k*th subcarrier is at the frequency:

(1) $$f_{k}=f_{c}+k/T,\;k=-K/2,\ldots ,K/2-1,$$

where $f_{c}$ is the carrier frequency. Assuming that there are *M* OFDM blocks in one frame, a passband waveform of the *m*th OFDM block at time $t^{\prime }\in \left[-T_{cp}+mT_{s},T+mT_{s}\right]$ is then given by

(2) $${\tilde{x}}^{\left(m\right)}\left(t\right)=2Re\left\{\frac{1}{\sqrt{K}}\sum_{k=-K/2}^{K/2-1}x_{k}^{\left(m\right)}e^{j2\pi f_{k}t}q\left(t\right)\right\},$$

where m=1,2,…,M, $t=t\prime -mT_{s}$, $x_{k}^{\left(m\right)}$ is defined as a transmitted symbol on the *k*th subcarrier at the *m*th OFDM block and q(t) is the pulse shaping filter:

(3) $$q\left(t\right)=\left\{\begin{array}{cc} 1 & ,\;t\in [-T_{cp},T],\\ 0 & ,\;otherwise. \end{array}\right.$$

The UWA channel is a typical sparse multipath channel. For horizontal shallow water multi-path transmission with a range much greater than depth, each path has almost the same Doppler factor [38]. Thus, we assume that all the paths have the equal Doppler scale factor *a*, and the Doppler effect can be almost eliminated through effective Doppler estimation and compensation. Furthermore, the path delay remains stable across several consecutive OFDM blocks, and the path gains and Doppler scale factors are constant during one OFDM block, but vary from block to block. The time-varying channel response during the *m*th OFDM block can be expressed as

(4) $$h^{\left(m\right)}\left(\tau ,t\right)=\sum_{l=0}^{L-1}A_{l}^{\left(m\right)}\delta \left[\tau -\left(\tau_{l}-a^{\left(m\right)}t\right)\right],$$

where *L* is the path number of the multipath channel, which may be distributed in more than one cluster. $A_{l}$ and $\tau_{l}$ denote the gain and delay of the *l*th path.

Through the time-varying channel, the received passband signal during the *m*th OFDM block can be expressed as

(5) $${\tilde{y}}^{\left(m\right)}\left(t\right)=\sum_{l=0}^{L-1}A_{l}^{\left(m\right)}{\tilde{x}}^{\left(m\right)}\left[\left(1+a^{\left(m\right)}\right)t-\tau_{l}\right]+{\tilde{w}}^{\left(m\right)}\left(t\right),$$

where $\tilde{w}\left(t\right)$ is the additive noise. After synchronization, a popular two-step approach [39] can be adopted to mitigate the Doppler effect, which takes a coarse Doppler estimation and resampling as the first step and then performs the fine Doppler shift compensation using the null subcarriers. Moreover, CP can also be used for Doppler shift estimation [40]. Performing CP-OFDM demodulation, the output K×1 vector of the *m*th OFDM block can be expressed as

(6) $$y^{\left(m\right)}=X^{\left(m\right)}Fh^{\left(m\right)}+w^{\left(m\right)},$$

where $X^{\left(m\right)}$ is an K×K diagonal matrix whose diagonal entries are the *K* transmitted symbols, F is an K×L discrete Fourier transform (DFT) matrix and $w^{\left(m\right)}$ is an K×1 additive noise vector. $h^{\left(m\right)}$ is the discretized CIR vector as $h^{\left(m\right)}=\left[h_{1}^{\left(m\right)},h_{2}^{\left(m\right)},\ldots ,h_{L}^{\left(m\right)}\right]$.

For *M* consecutive OFDM blocks, the transmission model of all K×M symbols can be expressed as

(7) $$Y=\left[\Phi^{\left(1\right)}h^{\left(1\right)},\Phi^{\left(2\right)}h^{\left(2\right)},\ldots ,\Phi^{\left(M\right)}h^{\left(M\right)}\right]+W,$$

where $Y=[y^{\left(1\right)},y^{\left(2\right)},\ldots ,y^{\left(M\right)}]$, $W=[w^{\left(1\right)},w^{\left(2\right)},\ldots ,w^{\left(M\right)}]$, and $H=[h^{\left(1\right)},h^{\left(2\right)},\ldots ,h^{\left(M\right)}]$. $\Phi^{\left(m\right)}$ is defined as $\Phi^{\left(m\right)}≜X^{\left(m\right)}F$. Considering the comb-type pilot arrangement, the transmission model of $K_{p}\times M$ pilot symbols can be expressed as

(8) $$Y_{P}=\Phi_{P}H+W_{P},$$

where $Y_{P}=\left[y_{p}^{\left(1\right)},y_{p}^{\left(2\right)},\ldots ,y_{p}^{\left(M\right)}\right]$ and $W_{P}=\left[w_{p}^{\left(1\right)},w_{p}^{\left(2\right)},\ldots ,w_{p}^{\left(M\right)}\right]$ are the submatrixs of Y and W at the location of the pilot subcarriers, respectively. $\Phi_{P}≜X_{P}F_{P}$ is the known dictionary matrix where $X_{P}$ is the $K_{p}\times K_{p}$ diagonal matrix with the known pilots along its diagonal and $F_{P}$ is the $K_{p}\times L$ DFT matrix.

By vectorizing H as $h=vec(H^{T})$, a pilot-based channel measurement model can be constructed according to (8), as

(9) $$y_{p}=U_{P}h+w_{p},$$

where $y_{p}=vec\left(Y_{P}^{T}\right)$, $w_{p}=vec\left(W_{P}^{T}\right)$ and $U_{P}=\Phi_{P}⊗I_{M}$. AWGN can be adopted to approximate the additive noise $w_{p}$. However, the attenuation and noise of the UWA channel may vary over the signal bandwidth. When a small number of pilot symbols are used to estimate the UWA channel with strong colored noise, the accuracy of the pilot-based channel measurement will decrease severely. To improve the performance of channel measurement, a noise-resistant channel measurement model is constructed according to (7) and it can be expressed as

(10) y=Uh+w,

where $y=vec(Y^{T})$, $w=vec(W^{T})$ and U is the KM×LM matrix as

(11) $$U=\left[\begin{array}{ccc} diag\{\Phi^{\left(1\right)}\left[1,1\right],\ldots ,\Phi^{\left(M\right)}\left[1,1\right]\} & \ldots & diag\{\Phi^{\left(1\right)}\left[1,L\right],\ldots ,\Phi^{\left(M\right)}\left[1,L\right]\}\\ \vdots & \ddots & \vdots \\ diag\{\Phi^{\left(1\right)}\left[K,1\right],\ldots ,\Phi^{\left(M\right)}\left[K,1\right]\} & \ldots & diag\{\Phi^{\left(1\right)}\left[K,L\right],\ldots ,\Phi^{\left(M\right)}\left[K,L\right]\} \end{array}\right],$$

where $\Phi^{\left(m\right)}\left[i,j\right]$ denotes the entry in the *i*th row and the *j*th column of $\Phi^{\left(m\right)}$. All subcarriers are used for channel measurement in (10), as the unknown data symbols can be obtained by data detection or decoding. Assuming that the power distribution over all subcarriers is non-uniform, $\lambda =[\lambda_{-K/2},\lambda_{-K/2+1},\ldots ,\lambda_{K/2-1}]$, w in (10) satisfies:

(12) w∼CN(0,Λ),

where $\Lambda =diag\left(\lambda \right)⊗I_{M}$.

### 3.3. Clustered-Sparse Channel Distribution Model

According to the measurement equation in (9) or (10), the channel vector h can be estimated. However, the UWA channel usually has an excessive multipath delay spread. With so many undetermined channel taps, the prior clustered-sparse structure of the UWA channel can be exploited to obtain the sparse solution of the channel vector. First, we assume all that the *L* paths (i.e., *L* rows) in H are mutually independent, which can form *C* clusters. There are $L_{d}$ paths in the *d*th cluster and these clusters do not overlap, each of which occupies dense path delays. With the same time correlation characteristics, the *d*th cluster satisfies a first-order auto-regressive (AR) model, given by

(13) $$H_{d}\left[i,j+1\right]=\beta_{d}H_{d}\left[i,j\right]+\sqrt{1-|\beta_{d}{|}^{2}}V_{d}\left[i,j\right],$$

where $\beta_{d}$ is the temporal correlation coefficient of the *d*th cluster. $H_{d}$ is a $L_{d}\times M$ submatrix of H, $i=1,2,\ldots ,L_{d}$ and j=1,2,…,M−1 are the row and column indices of $H_{d}$, respectively. $V_{d}$ is the model noise matrix with complex Gaussian distribution and assumed that $vec\left(V_{d}^{T}\right)∼\mathrm{CN}\left(0,\Gamma_{d}⊗I_{M}\right)$, where:

(14) $$\Gamma_{d}=diag\left(\gamma_{d,1},\gamma_{d,2},\ldots ,\gamma_{d,L_{d}}\right)$$

is a positive semi-definite diagonal real-valued matrix. Although a higher-order AR model may express better model approximation performance, it would lead to higher complexity. More importantly, a higher-order AR model means a higher overfitting risk, especially when there are only a few OFDM blocks in one frame. Hence, a first-order AR process was adopted to model the temporal correlation characteristic for each cluster of CIRs.

By vectorizing $H_{d}$ as $h_{d}=vec\left(H_{d}^{T}\right)$, the parameteric Bayesian prior of $h_{d}$ can be written by

(15) $$p\left(h_{d};\beta_{d},\gamma_{d}\right)=\mathrm{CN}\left(0,\Gamma_{d}⊗B_{d}\right),$$

where $\gamma_{d}=\left[\gamma_{d,1},\gamma_{d,2},\ldots ,\gamma_{d,L_{d}}\right]$ controls the sparsity of the $L_{d}$ paths in the *d*th cluster. When $Tr(\Gamma_{d})\to 0$, the associated $h_{d}\to 0$. In other words, $\Gamma_{d}$ reflects the probability density of the *d*th cluster. $B_{d}$ is a Toeplitz matrix with the following form:

(16) $$B_{d}=Toeplitz\left(1,\beta_{d},\ldots ,\beta_{d}^{M-1}\right),$$

and it reflects the variation characteristics of the *d*th cluster. $\Gamma_{d}$ and $B_{d}$ jointly determine the cluster distribution of the *d*th cluster. Accordingly, the prior probability density function of h can be written as

(17) $$p\left(h;\gamma_{1},\gamma_{2},\ldots ,\gamma_{C},\beta_{1},\beta_{2},\ldots ,\beta_{C}\right)=\mathrm{CN}\left(0,\sum_{0}\right),$$

where $\sum_{0}=diag\left(\Gamma_{1}⊗B_{1},\Gamma_{2}⊗B_{2},\ldots ,\Gamma_{C}⊗B_{C}\right)$.

## 4. Bayesian Learning-Based Clustered-Sparse Channel Estimation

In this section, the parametric form of Bayesian channel estimation is given first, and followed by the cluster distribution learning. Then, the partition-based clustered-sparse Bayesian learning algorithm is presented to obtain the clustered-sparse distribution and Bayesian channel estimate. At last, the complexity and performance analysis of this algorithm is given.

### 4.1. Bayesian Channel Estimation

To simplify the implementation complexity of Bayesian channel estimation, only known pilot symbols are used for channel measurement. As pilot symbols are sparsely distributed, it is difficult to estimate the non-uniform power spectrum at the location of pilot subcarriers. Therefore, the additive noise in (9) is approximated as white Gaussian noise with noise variance λ in this section. According to (9) and (17), using the Bayes rule, the posterior density of h can be obtained as

(18) $$p\left(h|y_{p};\lambda ,\gamma_{1},\gamma_{2}\ldots ,\gamma_{C},\beta_{1},\beta_{2},\ldots \beta_{C}\right)=\mathrm{CN}\left(\mu_{h},\sum_{h}\right),$$

which is the complex Gaussian distribution with the posterior mean and covariance matrix:

(19) $$\mu_{h}=\lambda^{-1}\sum_{h}U_{P}^{H}y_{p}=\sum_{0}U_{P}^{H}{\left(\lambda I+U_{P}\sum_{0}U_{P}^{H}\right)}^{-1}y_{p},$$

(20) $$\sum_{h}={\left(\sum_{0}^{-1}+\lambda^{-1}U_{P}^{H}U_{P}\right)}^{-1}=\sum_{0}-\sum_{0}U_{P}^{H}{\left(\lambda I+U_{P}\sum_{0}U_{P}^{H}\right)}^{-1}U_{P}\sum_{0}.$$

Obviously, the maximum posterior (MAP) estimates ${\hat{h}}_{MAP}≜\mu_{h}$ and the covariance matrix $\sum_{h}$ are related to the hyperparameters $\left[\lambda ,\gamma_{1},\gamma_{2},\ldots ,\gamma_{C},\beta_{1},\beta_{2},\ldots ,\beta_{C}\right]$. Using $K_{n}\times M$ null subcarriers $Y_{N}=\left[y_{n}^{\left(1\right)},\ldots ,y_{n}^{\left(M\right)}\right]$, λ can be estimated as

(21) $$\lambda =\frac{1}{K}E[∥\;y^{\left(m\right)}{∥}_{2}^{2}]\approx \frac{1}{MK_{n}}\sum_{j=1}^{M}\sum_{i=1}^{K_{n}}{\left|Y_{N}\left[i,j\right]\right|}^{2},$$

where *i* and *j* are the row and column indexes of $Y_{N}$, respectively. The remaining hyperparameters $\left[\gamma_{1},\gamma_{2},\ldots ,\gamma_{C},\beta_{1},\beta_{2},\ldots ,\beta_{C}\right]$ are related to the cluster distribution information.

### 4.2. Cluster Distribution Learning

The hyperparameters $\theta ≜\left[\gamma_{1},\gamma_{2},\ldots ,\gamma_{C},\beta_{1},\beta_{2},\ldots ,\beta_{C}\right]$ can be estimated by maximizing the marginal likelihood function $p(y_{p};\theta )$. This is equivalent to minimizing $-log\;p(y_{p};\theta )$, giving the effective cost function:

(22) $$L\left(\theta \right)=y_{p}^{H}\sum_{y}p^{-1}\left(\theta \right)y_{p}+log\left|\sum_{y}\left(\theta \right)\right|,$$

where $\sum_{y}p\left(\theta \right)=\lambda I+U_{P}\sum_{0}\left(\theta \right)U_{P}^{H}$. The above problem cannot be solved in closed form, and the expectation maximization (EM) method [41] can be employed to solve it iteratively. For unknown values of hyperparameters governing the prior density, h is considered as the nuisance variable and θ is estimated by maximizing:

(23) $$Q\left(\theta |\theta^{\left(old\right)}\right)=E_{h|y_{p};\theta^{\left(old\right)}}\left[log\;p\left(y_{p},h;\theta \right)\right]=E_{h|y_{p};\theta^{\left(old\right)}}\left[log\;p\left(y_{p}|h;\lambda \right)\right]+E_{h|y_{p};\theta^{\left(old\right)}}\left[log\;p\left(h;\theta \right)\right],$$

where $\theta^{\left(old\right)}$ denotes the estimated hyperparameters in the previous iteration. Ignoring the first term unrelated to θ, the Q Function (23) can be simplified to:

(24) $$\begin{array}{cc} Q(\beta_{1},\beta_{2},\ldots ,\beta_{C}, & \gamma_{1},\gamma_{2},\ldots ,\gamma_{C})=E_{h|y_{p};\theta^{\left(old\right)}}\left\{log\;p\left[h;B_{1},B_{2},\ldots ,B_{C},\Gamma_{1},\Gamma_{2},\ldots ,\Gamma_{C}\right]\right\}\\ \propto & -\frac{1}{2}\sum_{d=1}^{C}\left[log\;(|\Gamma_{d}{|}^{M}|B_{d}{|}^{L_{d}})+h_{d}^{H}\left(\Gamma_{d}^{-1}⊗B_{d}^{-1}\right)h_{d}\right]\\ \propto & -\frac{1}{2}\sum_{d=1}^{C}\left\{Mlog\;(|\Gamma_{d}\left|\right)+L_{d}log\;(|B_{d}\left|\right)+Tr\left[\left(\Gamma_{d}^{-1}⊗B_{d}^{-1}\right)\left(\sum_{h}^{d}+\mu_{h}^{d}{\left(\mu_{h}^{d}\right)}^{H}\right)\right]\right\} \end{array},$$

where $h_{d}\in C^{ML_{d}\times 1}$, $\mu_{h}^{d}\in C^{ML_{d}\times 1}$ and $\sum_{h}^{d}\in C^{ML_{d}\times ML_{d}}$ denote the corresponding *d*th cluster in $h\in C^{ML\times 1}$, $\mu_{h}\in C^{ML\times 1}$ and $\sum_{h}\in C^{ML\times ML}$, respectively. $\mu_{h}$ and $\sum_{h}$ are evaluated according to (19) and (20), given the estimated hyperparameters $\theta^{\left(old\right)}$ and the noise variance λ. As shown in (14) and (16), $\Gamma_{d}$ and $B_{d}$ are determined by the hyperparameters $\gamma_{d}$ and $\beta_{d}$, respectively.

The partial derivative of (24) with respect to $\gamma_{d,i}$$\left(d=1,2,\ldots ,C;i=1,2\ldots ,L_{d}\right)$ is given by

(25) $$\frac{\partial Q}{\partial \gamma_{d,i}}=-\frac{M}{2\gamma_{d,i}}+\frac{1}{2\gamma_{d,i}^{2}}Tr\left[B_{d}^{-1}\left(\sum_{h}^{d,i}+\mu_{h}^{d,i}{\left(\mu_{h}^{d,i}\right)}^{H}\right)\right],$$

where $\mu_{h}^{d,i}\in C^{M\times 1}$ denotes the corresponding *i*th path in $\mu_{h}^{d}$, and $\sum_{h}^{d,i}\in C^{M\times M}$ is the corresponding principal diagonal block for the *i*th path in $\sum_{h}^{d}$. So the learning rule for $\gamma_{d,i}$ can be given by

(26) $$\gamma_{d,i}=\frac{1}{M}Tr\left[B_{d}^{-1}\left(\sum_{h}^{d,i}+\mu_{h}^{d,i}{\left(\mu_{h}^{d,i}\right)}^{H}\right)\right].$$

The gradient of (24) over $B_{d}$(d=1,2,…,C) is:

(27) $$\frac{\partial Q}{\partial B_{d}}=-\frac{L_{d}}{2}B_{d}^{-1}+\frac{1}{2}\sum_{i=1}^{L_{d}}\frac{1}{\gamma_{d,i}}B_{d}^{-1}\left(\sum_{h}^{d,i}+\mu_{h}^{d,i}{\left(\mu_{h}^{d,i}\right)}^{H}\right)B_{d}^{-1}.$$

Thus, the learning rule for $B_{d}$ can be derived as

(28) $$B_{d}=\frac{1}{L_{d}}\sum_{i=1}^{L_{d}}\frac{\sum_{h}^{d,i}+\mu_{h}^{d,i}{\left(\mu_{h}^{d,i}\right)}^{H}}{\gamma_{d,i}}.$$

here, we constrain $B_{d}$ to have the form as shown in (16), and thus $\beta_{d}$ can be empirically calculated as $\beta_{d}≜\frac{\alpha_{1}}{\alpha_{0}}$ where $\alpha_{0}$ (resp. $\alpha_{1}$) is the average of the elements along the main diagonal (resp. the main sub-diagonal) of the matrix $B_{d}$ in (28).

### 4.3. Partition-Based Clustered-Sparse Bayesian Learning Algorithm

When the cluster partition information is known, the hyperparameters θ, the posterior mean $\mu_{h}$ and the posterior covariance $\sum_{h}$ can be iteratively obtained through the EM method. In other words, cluster partition and cluster distribution jointly determine the clustered-sparse channel distribution. However, it is usually difficult to obtain the cluster partition in advance. As shown in Figure 1, several significant clusters with high energy in CIRs are scattered over a long time spread, and usually do not overlap each other. As shown in (28), the boundary information of these clusters is important for calculating $B_{d}$, because different clusters have different coherence times (i.e., different AR coefficients). In particular, some weak clusters that can be ignored will cause the hyperparameter estimation error of significant clusters when these clusters are not distinguished. Therefore, the PB-CSBL algorithm is proposed to update cluster partition by pruning the paths with very small energy (i.e., $\gamma_{d,i}\to 0$). As shown in Algorithm 1, the PB-CSBL algorithm mainly includes three sub-parts: Channel Estimation, Cluster Partition, Cluster Evolution, and they will loop until the iteration stop condition is reached. In the sub-part of Cluster Partition, only the dense paths with high energy are retained to form clusters and the paths with negligible energy are pruned. For a cluster with some pruned paths, it can further split into more clusters according to the positions of the pruned paths. To reduce the impact of an inaccurate clustering structure at the beginning of the iteration, the maximum possible number of discrete paths in one cluster is given. In the sub-part of Cluster Evolution, the derived cluster distribution learning rules are included. A good initial estimation of the unknown hyperparameters is significant for achieving the global maximum instead of a local maximum. For practical implementation, it was found that an initial estimation given by $\Gamma_{d}=I_{L_{d}}$ and $B_{d}=I_{M}$ was sufficient for the proposed PB-CSBL algorithm.
**Algorithm 1:** Partition-Based Clustered-Sparse Bayesian Learning Algorithm**Input:** the received pilot signal $y_{p}$; the dictionary matrix $U_{P}$; the noise variance λ;

  the length of discrete paths *L*; the maximum number of iterations $r_{max}$;

  the maximum number of discrete paths in one cluster $LC_{max}$; the threshold for

  prunning small hyperparameters $\gamma_{th}$; the threshold to stop the whole

  algorithm ϵ.
**Initialize:** the list of path power $\gamma_{list}=1_{L}$; the number of clusters C=1;

  the list of cluster structure CLS : $\Gamma_{1}=I_{L}$, $B_{1}=I_{M}$ and the delay range of

  the 1st cluster $R_{d}=\left[0,L-1\right]$; the iteration counter r=0.
**Channel Estimation:**
 1: $\sum_{0}\leftarrow diag\left(\Gamma_{1}⊗B_{1},\Gamma_{2}⊗B_{2},\ldots ,\Gamma_{C}⊗B_{C}\right)$.

 2: $\sum_{h}=\sum_{0}-\sum_{0}U_{P}^{H}{\left(\lambda I+U_{P}\sum_{0}U_{P}^{H}\right)}^{-1}U_{P}\sum_{0}$.

 3: $\mu_{h}\leftarrow \lambda^{-1}\sum_{h}U_{P}^{H}y_{p}$.
**Cluster Evolution:**
 4: **for**
d=1,2,…,C**do**

 5:  **for** 
$i=1,2,\ldots ,L_{d}$
**do**

 6:   $\gamma_{d,i}\leftarrow \frac{1}{M}Tr\left[B_{d}^{-1}\left(\sum_{h}^{d,i}+\mu_{h}^{d,i}{\left(\mu_{h}^{d,i}\right)}^{H}\right)\right]$, and update $\gamma_{list}$ with $\gamma_{d,i}$.

 7:  **end for**

 8:  $L_{d}^{\prime }\leftarrow min\left(L_{d},LC_{max}\right)$.

 9:  $B_{d}\leftarrow \frac{1}{L_{d}^{\prime }}\sum_{i=1}^{L_{d}^{\prime }}\frac{\sum_{h}^{d,i}+\mu_{h}^{d,i}{\left(\mu_{h}^{d,i}\right)}^{H}}{\gamma_{d,i}}$ using the $L_{d}^{\prime }$ most significant continuous paths.

 10:  $\beta_{d}\leftarrow \frac{\alpha_{1}}{\alpha_{0}}$, where $\alpha_{1}$ and $\alpha_{0}$ can be obtained through $B_{d}$.

 11: **end for**
**Cluster Partition:**
 12: $p^{\left(0\right)}\leftarrow$ FindIndex$\left(0<\gamma_{list}<\gamma_{th}\right)$.

 13: **if**
$p^{\left(0\right)}$ is not empty **then**

 14:  $\gamma_{list}^{\left(old\right)}\leftarrow \gamma_{list}$ and $\gamma_{list}\left[p^{\left(0\right)}\right]\leftarrow 0$.

 15:  CLS← Split(CLS, $p^{\left(0\right)}$), where CLS is splitted into *C* clusters

    according to $p^{\left(0\right)}$.

 16:  $p^{\left(1\right)}\leftarrow$ FindIndex$\left(\gamma_{list}\geq \gamma_{th}\right)$ and $U_{P}\leftarrow U_{P}\left[:,p^{\left(1\right)}⊗\left(1:M\right)\right]$.

 17: **end if**

 18: **for**
d=1,2,…,C**do**

 19:  Update $R_{d}$, $\Gamma_{d}$ and $B_{d}$ in CLS according to $p^{\left(0\right)}$, $\gamma_{list}$ and $\beta_{d}$, respectively.

 20: **end for**
**Check stopping conditions:**
 21: r←r+1.

 22: **return** the sub-part of ChannelEstimation until $r\geq r_{max}$ or
   
$∥\gamma_{list}-\gamma_{list}^{\left(old\right)}{∥}_{2}^{2}<\epsilon$.
**Output:** the pruned channel $\mu_{h}$ with the covariance matrix $\sum_{h}$ and the

 cluster list CLS.


### 4.4. Complexity and Performance Analysis

Considering the excessive delay spread of the UWA channel and the purpose of saving pilot overhead, we assumed $K_{d}\geq L\geq K_{p}\geq M$. The time complexity of the proposed PB-CSBL algorithm is compared with the least squares (LS) [39], OMP [25], SOMP [27] and TMSBL [32]. In order to avoid involving matrix inversion, pilot subcarriers are equispaced in the LS method. For fair comparison, the residual Doppler expansion is ignored when evaluating the time complexity of the OMP method. The comparison of time complexity is shown in Table 1, where $\bar{N}$ denotes the average iteration number. It can be seen that the time complexity of the PB-CSBL algorithm is higher than that of the LS, OMP and SOMP. This is due to the fact that the temporal correlation of CIRs is ignored in the LS and OMP. Although the stability of path delays is exploited in the SOMP, the correlation of path gains is discarded. Therefore, LS, OMP and SOMP may exhibit worse channel estimation performance compared to the PB-CSBL algorithm. As for the TMSBL with $O(LK_{p}^{2})$ per iteration, it has lower time complexity than the proposed PB-CSBL with $O(LK_{p}^{2}M^{3})$ per iteration. This is mainly because it adopts the measure in [41], assuming that there is no temporal correlation among consecutive CIRs or no additive noise in received signal, but this may bring some performance loss, especially for slow-varying or high-noise channels. Therefore, when *M* is small, we do not adopt the simplification in [41]. The computational complexity of the PB-CSBL algorithm can be further reduced by implementing a first-order algorithm in E-step [34], but the implementation details are omitted here.

According to (20), the mean square error (MSE) bound of the sparse vector h can be expressed as

(29) $$E\left[∥h-\hat{h}{∥}_{2}^{2}\right]\geq Tr\left(\sum_{h}\right)=Tr\left[\sum_{0}-\sum_{0}U_{P}^{H}{\left(\lambda I+U_{P}\sum_{0}U_{P}^{H}\right)}^{-1}U_{P}\sum_{0}\right],$$

where $\hat{h}$ is the estimate of h, and its estimation accuracy is related to the hyperparameter vector θ obtained by maximizing the marginal likelihood function. Thus, the lower bound of MSE is reached (i.e., equality holds) only when the perfect hyperparameters are obtained.

Assume that all *L* paths are in one cluster (i.e., C=1) and consider two special cases of θ:

(1) The temporal correlation coefficient β=0.
  In this case, there is no temporal correlation among CIRs. It is assumed that each transmitted symbol is normalized to unit power. Then, the MSE bound of $h^{\left(m\right)}$ can be expressed by
  (30) $$E\left[∥h^{\left(m\right)}-{\hat{h}}^{\left(m\right)}{∥}_{2}^{2}\right]\geq Tr\left[{\left(\Gamma^{-1}+\lambda^{-1}\Phi_{P}^{H}\Phi_{P}\right)}^{-1}\right]=\sum_{l=1}^{L}{\left(\frac{K_{p}}{\lambda }+\frac{1}{\gamma_{l}}\right)}^{-1},$$
  where Γ=diag(γ), $\gamma =[\gamma_{1},\gamma_{2},\ldots ,\gamma_{L}]$ and $\gamma_{l}$ controls the variance of the *l*th channel coefficient.
(2) The temporal correlation coefficient β=1
  In this case, the CIRs are time-invariant across *M* OFDM blocks. Thus, the additive noise variance λ can be reduced to λ/M, and the MSE bound of $h^{\left(m\right)}$ can be expressed by
  (31) $$E\left[∥h^{\left(m\right)}-{\hat{h}}^{\left(m\right)}{∥}_{2}^{2}\right]\geq \sum_{l=1}^{L}{\left(\frac{MK_{p}}{\lambda }+\frac{1}{\gamma_{l}}\right)}^{-1}.$$

As shown in the case of the high temporal correlation (i.e., β=1), the anti-noise performance of channel estimation can be improved. As for the low temporal correlation among CIRs (i.e., β=0), the anti-noise performance is not significantly improved. However, with more reference information, the better hyperparameter vector γ can be obtained. Therefore, when multiple OFDM blocks are utilized simultaneously, higher channel estimation accuracy can be achieved.

## 5. Noise-Corrected Clustered-Sparse Channel Estimation

In this section, the parametric form of noise-resistant Bayesian channel estimation is given first. Then, the data detection and noise measurement are derived. Then, the noise-corrected clustered-sparse channel estimation algorithm is presented. At last, the complexity and performance analysis of this algorithm is given.

### 5.1. Noise-Resistant Bayesian Channel Estimation

As the number of pilots $K_{p}$ decreases, the precision of channel estimation using the PB-CSBL algorithm will rapidly deteriorate. This is primarily because the algorithm does not utilize all the information available. Specifically, although both pilot and data symbols are transmitted, only the $K_{p}\times M$ matrix $Y_{P}$ corresponding to the pilot subcarriers in (8) is used for estimating the CIRs. For the matrix Y in (7), the remaining $K_{d}\times M$ observations are typically not used as they contain the unknown data symbols. If the unknown data symbols are perfectly obtained, using the Bayes rule, the posterior mean of CIRs based on the prior probability in (17) and the channel measurement equation in (10) can be written by

(32) $${\bar{\mu }}_{h}=\sum_{0}U^{H}{\left(\Lambda +U\sum_{0}U^{H}\right)}^{-1}y,$$

with the posterior covariance matrix:

(33) $${\bar{\sum }}_{h}=\sum_{0}-\sum_{0}U^{H}{\left(\Lambda +U\sum_{0}U^{H}\right)}^{-1}U\sum_{0}.$$

Moreover, AWGN is assumed to simplify implementation complexity in the PB-CSBL algorithm, as the noise power distribution $\lambda =[\lambda_{-K/2},\lambda_{-K/2+1},\ldots ,\lambda_{K/2-1}]$ depends on the observation of all subcarriers. However, in a complex UWA environment, the noise power may vary significantly over the signal bandwidth. Using decided data symbols and corrected colored noise, the high estimation accuracy can be achieved by the MAP estimate in (32).

### 5.2. Data Detection and Noise Measurement

To obtain the MAP estimate in (32), the hyperparameters related to the cluster distribution, $\theta ≜\left[\gamma_{1},\gamma_{2},\ldots ,\gamma_{C},\beta_{1},\beta_{2},\ldots ,\beta_{C}\right]$, can be iteratively updated, as shown in the proposed PB-CSBL algorithm, however, the detection of data signal $x_{d}^{\left(m\right)}$ and the noise distribution λ are still unresolved. Using the notations $x_{d}≜\left[{\left(x_{d}^{\left(1\right)}\right)}^{T},{\left(x_{d}^{\left(2\right)}\right)}^{T},\ldots ,{\left(x_{d}^{\left(M\right)}\right)}^{T}\right]$, $\varphi ≜\left[\lambda ,x_{d}\right]$, and $\zeta ≜\left[\theta ,\varphi \right]$, the unknown hyperparameters set ζ can be estimated by maximizing the marginal likelihood function p(y;ζ). This is equivalent to minimizing −logp(y;ζ), giving the effective cost function:

(34) $$L\left(\zeta \right)=y^{H}\sum_{y}^{-1}\left(\zeta \right)y+log\left|\sum_{y}\left(\zeta \right)\right|,$$

where $\sum_{y}\left(\zeta \right)=\Lambda \left(\lambda \right)+U\left(x_{d}\right)\sum_{0}\left(\theta \right)U^{H}\left(x_{d}\right)$. Similar to the cluster distribution learning, the expectation maximization (EM) method [41] can be employed to solve (34) iteratively. While h is considered as the nuisance variable, ζ can be estimated by maximizing:

(35) $$Q\left(\zeta |\zeta^{\left(old\right)}\right)=E_{h|y;\zeta^{\left(old\right)}}\left[log\;p\left(y,h;\zeta \right)\right]=E_{h|y;\zeta^{\left(old\right)}}\left[log\;p\left(y|h;\varphi \right)\right]+E_{h|y;\zeta^{\left(old\right)}}\left[log\;p\left(h;\theta \right)\right],$$

where $\zeta^{\left(old\right)}$ denotes the estimated hyperparameters in the previous iteration. As shown in (35), the joint maximization simplifies into two independent maximizations over θ and φ. On optimizing the latter function in (35) with respect to θ, one obtains the learning rules about θ as (26) and (28). Maximizing the former over $x_{d}^{\left(m\right)}$ yields the minimum mean squared error (MMSE) equalizer:

(36) $${\hat{x}}_{d}^{\left(m\right)}={\left[diag\left(\lambda_{d}\right)C_{X}^{-1}+Prdiag\left(F_{D}\Xi_{h}^{\left(m\right)}F_{D}^{H}\right)\right]}^{-1}diag\left[{\left({\bar{\mu }}_{h}^{\left(m\right)}\right)}^{H}F_{D}^{H}\right]y_{d}^{\left(m\right)},$$

where $y_{d}^{\left(m\right)}$ and $\lambda_{d}$ are the subvectors of $y^{\left(m\right)}$ and λ at the location of data subcarriers, respectively. $C_{X}$ is the covariance matrix of $x_{d}^{\left(m\right)}$, $F_{D}$ is the $K_{d}\times L$ DFT matrix at the location of data subcarriers. $\Xi_{h}^{\left(m\right)}$ is defined as $\Xi_{h}^{\left(m\right)}≜{\bar{\mu }}_{h}^{\left(m\right)}{\left({\bar{\mu }}_{h}^{\left(m\right)}\right)}^{H}+{\bar{\sum }}_{h}^{\left(m\right)}$, where ${\bar{\mu }}_{h}^{\left(m\right)}\in C^{L\times 1}$ and ${\bar{\sum }}_{h}^{\left(m\right)}\in C^{L\times L}$ denote the posterior mean and covariance matrix of the *m*th OFDM block, respectively. The decisions on the data symbols, $x_{d}^{\left(m\right)}$, are obtained by mapping the soft decisions, ${\hat{x}}_{d}^{\left(m\right)}$, to the closest point on the constellation, meaning that:

(37) $$x_{d}^{\left(m\right)}=Quantization\left({\hat{x}}_{d}^{\left(m\right)}\right).$$

Although the noise distribution is non-uniform, the noise powers of adjacent subcarriers are usually close (especially when the number of subcarriers is large). Then, we divide all subcarriers into non-overlapping *Q* groups, so that K/Q subcarriers can be grouped into the same one. Thus, the average noise power of the *q*th group can be calculated as

(38) $$\lambda_{g}^{\left(q\right)}=\frac{Q}{KM}Tr\left[y^{\left(q\right)}{\left(y^{\left(q\right)}\right)}^{H}-U^{\left(q\right)}\Xi_{h}{\left(U^{\left(q\right)}\right)}^{H}\right],$$

where $\Xi_{h}$ is defined as $\Xi_{h}≜{\bar{\mu }}_{h}{\left({\bar{\mu }}_{h}\right)}^{H}+{\bar{\sum }}_{h}$. $U\in C^{KM\times LM}$ can be calculated according to (11), as unknown data symbols are estimated by (36) and (37). $U^{\left(q\right)}\in C^{\frac{KM}{Q}\times LM}$ is the submatrix of U and denotes the *q*th group. $y^{\left(q\right)}$ denotes the *q*th group of y. After $\lambda_{g}^{\left(1\right)},\lambda_{g}^{\left(2\right)},\ldots ,\lambda_{g}^{\left(Q\right)}$ are calculated according to (38), the non-uniform noise power distribution across all subcarriers, $\lambda =[\lambda_{-K/2},\lambda_{-K/2+1},\ldots ,\lambda_{K/2-1}]$, can be obtained by upsampling the *Q* average noise powers.

### 5.3. Noise-Corrected Clustered-Sparse Channel Estimation Algorithm

Based on the noise-resistant Bayesian channel estimation, noise measurement and data detection, we can develop the PB-CSBL algorithm into the NC-CSCE Algorithm. As shown in Algorithm 2, the sub-parts of Cluster Partition and Cluster Evolution have the similar process to the PB-CSBL algorithm. With the aid of Data Detection and Noise Measurement, the estimation accuracy in Channel Estimation is improved. Conversely, the improvement of channel estimation also ensures the high accuracy of data detection and noise measurement results. To achieve the global maximum instead of a local maximum, the NC-CSCE algorithm requires a good initial estimate with respect to the unknown cluster distribution, data signal and noise distribution. However, it is obviously difficult to initialize so many hyperparameters at the same time. Therefore, the PB-CSBL algorithm is utilized to initialize ${\bar{\mu }}_{h}$ and ${\bar{\sum }}_{h}$ instead of initializing these hyperparameters. Once ${\bar{\mu }}_{h}$ and ${\bar{\sum }}_{h}$ are initialized, these hyperparameters can be calculated as shown in Algorithm 2.

### 5.4. Complexity and Performance Analysis

The NC-CSCE algorithm utilizes all available subcarriers to achieve better channel estimation performance. Therefore, compared with the proposed PB-CSBL algorithm with $O(LK_{p}^{2}M^{3})$ per iteration, the NC-CSCE algorithm has higher time complexity, i.e., $O({\left(K_{d}+K_{p}\right)}^{3}M^{3})$ per iteration. Similar to the PB-CSBL algorithm, the complexity of the NC-CSCE algorithm can be further simplified by using the techniques in [34,41], however, these simplifications may lead to the performance degradation of channel estimation because of the errors introduced by the approximations. The details of the simplifications are omitted here.
**Algorithm 2:** Noise-Corrected Clustered-Sparse Channel Estimation Algorithm**Input:** the received signal y; the noise variance λ; the length of discreted paths *L*;

  the maximum number of iterations $r_{max}$; the maximun number of discreted

  paths in one cluster $LC_{max}$; the threshold for prunning small

  hyperparameters $\gamma_{th}$; the threshold to stop the whole algorithm ϵ.
**Initialize:** The PB-CSBL algorithm is utilized to obtain the posterior mean ${\bar{\mu }}_{h}\leftarrow \mu_{h}$

  and covariance matrix ${\bar{\sum }}_{h}\leftarrow \sum_{h}$; the noise vector $\lambda =\lambda I_{K}$; the list of path

  power $\gamma_{list}=1_{L}$; the iteration counter r=0.
**Cluster Evolution:**
 1: **for**
d=1,2,…,C**do**

 2:  **for**
$i=1,2,\ldots ,L_{d}$
**do**

 3:   $\gamma_{d,i}\leftarrow \frac{1}{M}Tr\left[B_{d}^{-1}\left({\bar{\sum }}_{h}^{d,i}+{\bar{\mu }}_{h}^{d,i}{\left({\bar{\mu }}_{h}^{d,i}\right)}^{H}\right)\right]$, and update $\gamma_{list}$ with $\gamma_{d,i}$.

 4:  **end for**

 5:  $L_{d}^{\prime }\leftarrow min\left(L_{d},LC_{max}\right)$.

 6:  $B_{d}\leftarrow \frac{1}{L_{d}^{\prime }}\sum_{i=1}^{L_{d}^{\prime }}\frac{{\bar{\sum }}_{h}^{d,i}+{\bar{\mu }}_{h}^{d,i}{\left({\bar{\mu }}_{h}^{d,i}\right)}^{H}}{\gamma_{d,i}}$ using the $L_{d}^{\prime }$ most significant continuous paths.

 7:  $\beta_{d}\leftarrow \frac{\alpha_{1}}{\alpha_{0}}$, where $\alpha_{1}$ and $\alpha_{0}$ can be obtained through $B_{d}$.

 8: **end for**
**Cluster Partition:**
 9: $p^{\left(0\right)}\leftarrow$ FindIndex$\left(0<\gamma_{list}<\gamma_{th}\right)$.

 10: **if**$p^{\left(0\right)}$ is not empty **then**

 11:  $\gamma_{list}^{\left(old\right)}\leftarrow \gamma_{list}$ and $\gamma_{list}\left[p^{\left(0\right)}\right]\leftarrow 0$.

 12:  CLS← Split(CLS, $p^{\left(0\right)}$), where CLS is splitted into *C* clusters according

  to $p^{\left(0\right)}$.

 13: **end if**

 14: **for**
d=1,2,…,C**do**

 15:  Update $R_{d}$, $\Gamma_{d}$ and $B_{d}$ in CLS according to $p^{\left(0\right)}$, $\gamma_{list}$ and $\beta_{d}$, respectively.

 16: **end for**
**Data Detection:**
 17: Refer to (36) and (37).
**Noise Measurement:**
 18: Update the noise vector λ according to (38).
**Channel Estimation:**
 19: $\sum_{0}\leftarrow diag\left(\Gamma_{1}⊗B_{1},\Gamma_{2}⊗B_{2},\ldots ,\Gamma_{C}⊗B_{C}\right)$.

 20: Obtain U according to (11).

 21: $p^{\left(1\right)}\leftarrow$ FindIndex$\left(\gamma_{list}\geq \gamma_{th}\right)$ and $U\leftarrow U[:,p^{\left(1\right)}⊗\left(1:M\right)]$.

 22: Refer to (32) and (33).
**Check stopping conditions:**
 23: r←r+1.

 24: **return** the sub-part of ClusterEvolution until $r\geq r_{max}$ or $∥\gamma_{list}-\gamma_{list}^{\left(old\right)}{∥}_{2}^{2}<\epsilon$.
**Output:** the pruned channel ${\bar{\mu }}_{h}$ with the covariance matrix ${\bar{\sum }}_{h}$, the cluster list CLS,

  the noise vector λ, and the transmitted data symbols $x_{d}^{\left(m\right)}$ for m=1,2,…,M.


Based on the noise-resistant Bayesian channel estimation in the NC-CSCE algorithm, the MSE bound of the sparse vector h can be expressed as

(39) $$E\left[∥h-\hat{\bar{h}}{∥}_{2}^{2}\right]\geq Tr\left({\bar{\sum }}_{h}\right)=Tr\left[\sum_{0}-\sum_{0}U^{H}{\left(\Lambda +U\sum_{0}U^{H}\right)}^{-1}U\sum_{0}\right],$$

where $\hat{\bar{h}}$ is the estimate of h. The MSE bound is related to the hyperparameters of the cluster distribution, data symbols and noise distribution. Only when the perfect hyperparameters are obtained is the lower bound of MSE reached. Similarly to analyzing the PB-CSBL algorithm, we assume that all *L* paths are in one cluster (i.e., C=1) and consider the two special cases, namely the temporal correlation coefficient β=0 and β=1. For ease of analysis, the noise power keeps constant $\bar{\lambda }$ over all subcarriers. Furthermore, each transmitted symbol is normalized to unit power. Then, the MSE bounds of $h^{\left(m\right)}$ can be evaluated by

(40) $$E\left[∥h^{\left(m\right)}-{\hat{\bar{h}}}^{\left(m\right)}{∥}_{2}^{2}\right]\geq \sum_{l=1}^{L}{\left(\frac{K_{p}+K_{d}}{\bar{\lambda }}+\frac{1}{\gamma_{l}}\right)}^{-1},$$

(41) $$E\left[∥h^{\left(m\right)}-{\hat{\bar{h}}}^{\left(m\right)}{∥}_{2}^{2}\right]\geq \sum_{l=1}^{L}{\left(\frac{M(K_{p}+K_{d})}{\bar{\lambda }}+\frac{1}{\gamma_{l}}\right)}^{-1},$$

respectively. In addition to what has been discussed when analyzing the PB-CSBL algorithm, Equations (40) and (41) also prove that the NC-CSCE algorithm utilizing pilot symbols and unknown data symbols can achieve a smaller MSE compared with the PB-CSBL algorithm.

## 6. Evaluation and Result Analysis

In this section, to evaluate the performance of the proposed algorithms, numerical simulations and a lake trial were conducted. The parameter settings of CP-OFDM in the simulations and lake trial are shown in Table 2. The bandwidth, carrier frequency and sampling frequency of the lake trial are closely related to the transducers used in the trial, and these values are set smaller in the simulation system to increase the simulation speed. Because of the high cost of the lake trial, the CP length, the number of pilot subcarriers and the number of null subcarriers are designed to be larger than the simulation system to ensure the availability of trial data. In the simulation system (the specifications of our computer are as follows: Intel(R) Core(TM) i5-6400 CPU 2.7 GHz (4 cores), 16 GB RAM, 1 TB memory with Windows and MATLAB installed), it is assumed that the UWA channel has 12 randomly generated paths, and that these paths remain fixed within one OFDM frame. The maximum path delay is less than CP length, and the amplitudes of paths are Rayleigh distributed with the average power decreasing exponentially with delay. The Doppler scale factor $a\in (0,10^{-3})$ is randomly chosen. Moreover, there is no channel code module in the simulation system.

### 6.1. Simulation Results

Firstly, the proposed PB-CSBL and NC-CSCE algorithms were compared with the LS, OMP, SOMP and TMSBL for channel estimation. All paths are distributed in three non-overlapping clusters, and these clusters are set with three different temporal correlation coefficients ranges over multiple OFDM blocks, which are [0.1,0.3], [0.5,0.7], [0.8,1.0]. The additive noise is assumed to be white Gaussian noise, and thus all subcarriers are divided into the same group (i.e., Q=1) for the proposed NC-CSCE algorithm. The transmitted data bits are mapped to QPSK or 16-QAM constellation symbols. The performance metrics used in the numerical simulations are the MSE of CIRs and BER of data bits, which are verified in 500 simulations for each value of SNR.

**Figure 3:**
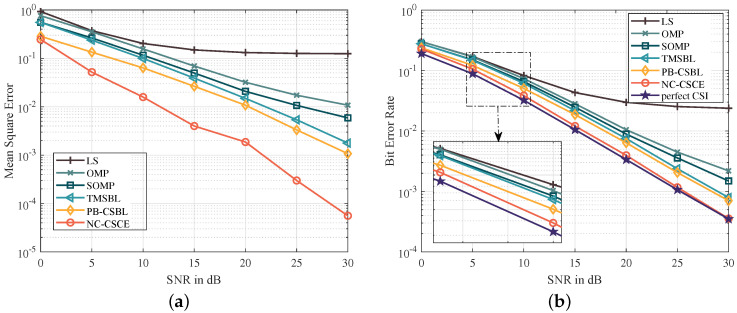
The comparison of MSE and BER performance based on QPSK constellation mapping: (**a**) the comparison of MSE performance; and (**b**) the comparison of BER performance.

**Figure 4:**
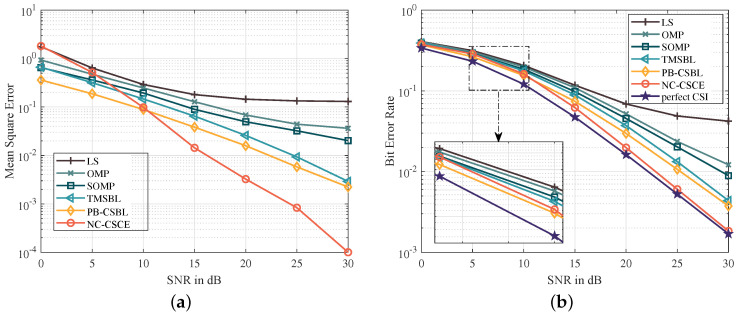
The comparison of MSE and BER performance based on 16-QAM constellation mapping: (**a**) the comparison of MSE performance; and (**b**) the comparison of BER performance.

Figure 3a,b show the MSE and BER performance of different channel estimation methods, respectively. LS performs worst, because the channel sparsity is not considered for this underdetermined system. Based on the joint estimation of multiple OFDM blocks, the SOMP makes full use of the strong temporal correlation of path delays and achieves better performance than the OMP. The delay and correlation of path gains are analyzed in the TMSBL, which makes it better than the SOMP. However, the proposed PB-CSBL performs even better, as it can learn different temporal correlation coefficients and reduce the interference among clusters. The proposed NC-CSCE algorithm utilizes all received pilot and data symbols for the channel estimation, which achieves the lowest BER close to the perfect CSI. When 16-QAM mapping is adopted, Figure 4 proves that the proposed algorithms still maintain good estimation performance. However, in the low SNR region from 0 to 10 dB, the NC-CSCE algorithm does not perform well. This is mainly because high-order modulation at low SNR is prone to symbol decision errors, which may be exacerbated in the iterative process of the NC-CSCE algorithm.

**Figure 5:**
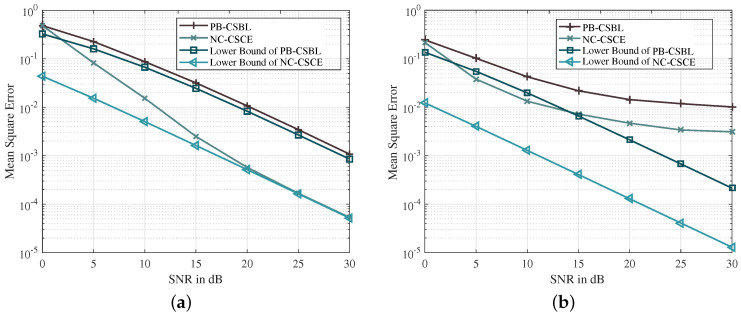
The MSE performance of the proposed algorithms and their theoretical lower bounds: (**a**) the temporal correlation coefficient β=0; and (**b**) the temporal correlation coefficient β=1.

**Figure 6:**
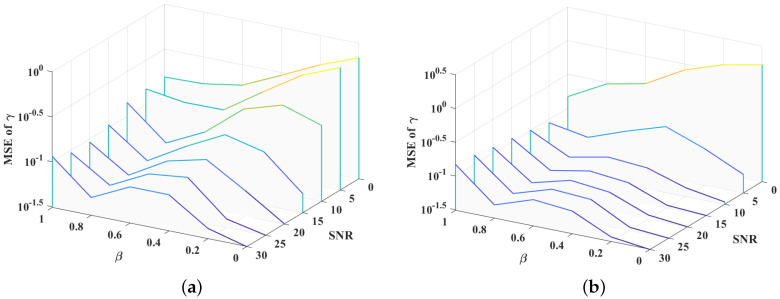
The MSE of the estimated hyperparameter γ by the proposed PB-CSBL and NC-CSCE algorithms under different SNRs and temporal correlation coefficients: (**a**) the PB-CSBL algorithm; and (**b**) the NC-CSCE algorithm.

Then, to verify the estimation performance of the proposed algorithms under extreme temporal correlation coefficients (i.e., β=0 and β=1), the proposed algorithms are compared with their theoretical lower bounds. Multipaths are assumed to be sparse and share the same correlation coefficient. Then, under QPSK mapping and white Gaussian noise, the mean square error performance is presented in Figure 5. When β=0, the MSE of the proposed algorithms is close to the theoretical lower bound in a high SNR region. A significant gap exists in the low SNR region, especially for the NC-CSCE algorithm. This is caused by imperfect hyperparameter estimation, as the lower bound is only reached when the perfect hyperparameters are obtained. When β=1, the gap is more obvious in the whole SNR region because of the hyperparameter estimation error. However, the MSE performance with β=1 is better than the MSE performance with β=0 in a low SNR region. In short, a larger temporal correlation coefficient means stronger resistance to additive noise, however, the hyperparameter estimation error, such as the error of γ, is often larger than that of small temporal correlation coefficients in the high SNR region. Figure 6a,b show the MSE of the γ estimated by the proposed PB-CSBL and NC-CSCE algorithms, respectively. The MSE is evaluated under different SNRs and temporal correlation coefficients. As can be seen, a larger temporal correlation coefficient achieves a smaller MSE in the low SNR region, while a smaller temporal correlation coefficient reduces the MSE value in the high SNR region.

**Figure 7:**
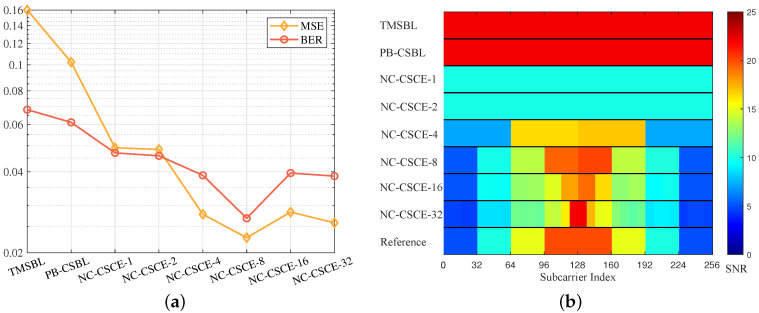
The performance comparison of different algorithms under colored noise: (**a**) the MSE and BER performance; and (**b**) the estimated SNRs over all subcarriers.

Finally, to evaluate the estimation performance of the proposed NC-CSCE algorithm under colored noise, we compared the NC-CSCE algorithm with the TMSBL and PB-CSBL algorithms. The group number of subcarriers in NC-CSCE is adjusted to evaluate the channel estimation performance. In the simulation, all paths are distributed in three non-overlapping clusters. The clusters are set with three different temporal correlation coefficients ranges over multiple OFDM blocks, which are [0.1,0.3], [0.5,0.7], [0.8,1.0]. All subcarriers are divided into eight groups and each group is set with a different SNR. As shown in Figure 7b, the reference SNR ranges are from 5 to 20 dB. With QPSK mapping, the MSE and BER performance curves are shown in Figure 7a, and the results prove that the proposed NC-CSCE algorithm is superior to the TMSBL and PB-CSBL algorithms. The NC-CSCE-8 denotes the NC-CSCE algorithm with eight groups, and it performs better than the NC-CSCE algorithm with other groups. Therefore, the setting of group number is critical for the NC-CSCE algorithm. In one instance, the actual distribution of colored noise can hardly be reflected with a limited group number; yet in another, a small symbol number will result in low measurement accuracy in one group. Figure 7b also shows the statistical results of the estimated SNRs based on 500 simulations. Similarly, NC-CSCE-8 matches the reference best among all compared methods.

### 6.2. Lake Experimental Results

To further verify the proposed algorithms, a real lake experiment was performed at Huating Lake, Anhui Province, China, in May 2018. Huating Lake covers an area of about 70 square kilometers, with an average water depth of about 17 m. We hung the transmitter and receiver on the side of a passenger ship and a speedboat, respectively. The passenger ship has a semi-open roof and is about 10 m long, while the speedboat is about half the size of the ship. During the experiment, the transmitter depth was about 10 m and the receiver depth was about 13 m. Affected by wind and current, their relative speed was about 0.5 m/s, and the distance between them was more than 4.5 km. The parameter settings of the experiment are in Table 2. In addition, the linear frequency modulation (LFM) signal was added at the beginning and end of each frame for time synchronization and Doppler estimation. The transmitted data bits were encoded by a 1/2 rate convolutional code, and the encoded bits were mapped by QPSK constellation. As showed in Figure 1, using the proposed NC-CSCE algorithm, we obtained the estimated CIRs for a 14 frame history with five consecutive OFDM blocks in one frame. The CIRs are estimated after Doppler compensation, and the total delay spread is around 15 ms. From the estimated CIRs, we notice that the paths are distributed in multiple clusters, and the channel shows a clustered-sparse structure.

**Figure 8:**
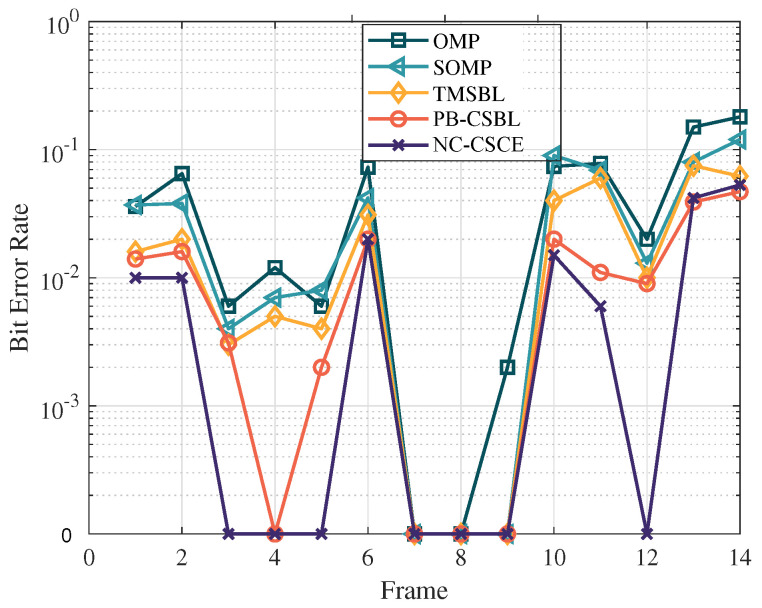
The comparison of BER performance in the lake experiment.

Figure 8 shows the BER performance after convolutional decoding. The BERs of the proposed two algorithms are still the lowest for most frames, followed by the TMSBL and the SOMP, while the OMP has the worst BER performance. Obviously, the performance gap between proposed algorithms and other methods further verify the superior performance of the proposed algorithms.

## 7. Conclusions

In this paper, we propose a novel Bayesian learning-based channel estimation architecture to estimate the time-varying multipath channel with colored noise for UWA-OFDM systems. Specifically, a clustered-sparse channel distribution model was constructed to characterize the multipath distribution, and a noise-resistant channel measurement model is constructed to reduce the noise disturbance. To obtain the clustered-sparse distribution, we propose the partition-based clustered-sparse Bayesian learning algorithm. To lessen the effect of colored noise, we proposed a noise-corrected clustered-sparse channel estimation algorithm to improve the estimation performance. Taking advantage of the iterative clustered-sparse distribution learning, symbol decision and noise correction, the accuracy of Bayesian channel estimation can be improved. Experiments proved the effectiveness of the proposed algorithms for channel estimation.

In future work, channel decoding can be incorporated into the Bayesian learning-based channel estimation architecture to explore the possibility of reducing symbol decision errors. In the noise-resistant channel measurement model, the channel gains are approximately constant within one OFDM block, which may be too optimistic in some rapidly time-varying UWA channels. Therefore, some channel estimation models that deal with doubly selective fading channels, such as the popular basis expansion model, can be combined with the clustered-sparse Bayesian learning to improve the estimation performance. At the same time, channel feedback and reconstruction will be analyzed to realize adaptive modulation and coding in UWA-OFDM communication.

## Figures and Tables

**Table 1:** The comparison of time complexity.

LS	OMP	SOMP	TMSBL	PB-CSBL
$O(LK_{p}M)$	$O(L^{2}K_{p}M)$	$O(L^{2}K_{p}M)$	$O(LK_{p}^{2}\bar{N})$	$O(LK_{p}^{2}M^{3}\bar{N})$

**Table 2:** UWA CP-OFDM settings.

Parameters	Notations	Values of the Simulations	Values of the Lake Trial
Bandwidth	*B*	1.6 kHz	5 kHz
Carrier frequency	$f_{c}$	2.5 kHz	20 kHz
Sampling frequency	$f_{s}$	12.5 kHz	100 kHz
Number of subcarriers	*K*	256	256
Number of data subcarriers	$K_{d}$	218	193
Number of pilot subcarriers	$K_{p}$	14	32
Number of null subcarriers	$K_{n}$	24	31
Symbol duration without CP	*T*	160 ms	51.2 ms
CP length	$T_{cp}$	10 ms	25.6 ms
Blocks in one frame	*M*	4	5

## Data Availability

Not applicable.
